# Retraction Note: The JAK2/STAT3 and mitochondrial pathways are essential for quercetin nanoliposome-induced C6 glioma cell death

**DOI:** 10.1038/s41419-025-07654-5

**Published:** 2025-04-15

**Authors:** G. Wang, J. J. Wang, X. L. Chen, S. M. Du, D. S. Li, Z. J. Pei, H. Lan, L. B. Wu

**Affiliations:** 1 Department of Pharmacy, Taihe Hospital, Hubei University of Medicine, Shiyan City, Hubei Province People’s Republic of China; 2 Hubei Provincial Key Laboratory of Embryo Stem Cells, Shiyan City, Hubei Province People’s Republic of China

Retraction to: *Cell Death and Disease* 10.1038/cddis.2013.242, published online 01 August 2013

The Editors-in-Chief have retracted the article. Multiple image concerns were raised including similarities across figures 1A-C, within figure 1D, and within figure 4, as well as with a paper published previously by the same authors [1]. Two panels from figure 1D and 1E were also found to be included in figure 4E of a paper published a few months before by the same authors, representing different conditions [2]. The Editors-in-Chief have therefore lost confidence in the underlying data of this article.

Authors X. L. Chen, D. S. Li, Z. J. Pei, H. Lan & L. B. Wu could not be contacted by the publisher. The first author provided an unsatisfactory response to the concerns raised but did not respond to correspondence regarding this retraction notice. The corresponding author has not responded to any correspondence from the publisher regarding this retraction.
